# Nicotinamide mononucleotide attenuates HIF-1α activation and fibrosis in hypoxic adipose tissue *via* NAD^+^/SIRT1 axis

**DOI:** 10.3389/fendo.2023.1099134

**Published:** 2023-01-26

**Authors:** Keke Wu, Biao Li, Yingxu Ma, Tao Tu, Qiuzhen Lin, Jiayi Zhu, Yong Zhou, Na Liu, Qiming Liu

**Affiliations:** ^1^ Department of Cardiovascular Medicine, Second Xiangya Hospital, Central South University, Changsha, Hunan, China; ^2^ Department of Cardiology, Shenzhen Traditional Chinese Medicine Hospital, Shenzhen, Guangdong, China

**Keywords:** nicotinamide mononucleotide, adipose tissue fibrosis, inflammation, HIF-1α activation, NAD +/SIRT1 axis

## Abstract

**Background:**

Fibrosis is increasingly considered as a major contributor in adipose tissue dysfunction. Hypoxic activation of hypoxia-inducible factor 1α (HIF-1α) induces a profibrotic transcription, leading to adipose fibrosis. Nicotinamide mononucleotide (NMN), a member of the vitamin B_3_ family, has been shown to relieve hepatic and cardiac fibrosis, but its effects on hypoxic adipose fibrosis and the underlying mechanism remain unclear. We aimed to elucidate the roles of NMN in regulating HIF-1α and fibrosis in hypoxic adipose tissue.

**Methods:**

Mice were placed in a hypobaric chamber for four weeks to induce adipose fibrosis. NMN (500 mg/kg, every three days) was administered by intraperitoneal injection. *In vitro*, Stromal vascular fractions (SVF) cells were treated by hypoxia with or without NMN (200μM), sirtinol (25μM, a SIRT1 inhibitor) and CoCl_2_ (100μM, a HIF1α enhancer). The effects of NMN on hypoxia-associated adipose fibrosis, inflammation, NAD^+^/SIRT1 axis alteration, and HIF-1α activation were evaluated by real-time polymerase chain reaction (PCR), western blots, immunohistochemistry staining, immunoprecipitation, and assay kits.

**Results:**

Mice placed in a hypoxic chamber for four weeks showed obvious adipose fibrosis and inflammation, which were attenuated by NMN. NMN also restore the compromised NAD^+^/SIRT1 axis and inhibited the activation of HIF-1α induced by hypoxia. In hypoxia-induced SVFs, the SIRT1 inhibitor sirtinol blocked the anti-fibrotic and anti-inflammatory effects of NMN, upregulated the HIF-1α and its acetylation level. The HIF1α stabilizer CoCl_2_ showed similar effects as sirtinol.

**Conclusion:**

NMN effectively attenuated HIF-1α activation-induced adipose fibrosis and inflammation by restoring the compromised NAD^+^/SIRT1 axis.

## Introduction

Obesity is commonly considered as a persistent low-level inflammatory state closely related to insulin resistance, metabolic syndromes, and cardiovascular diseases (1, 2). This inflammatory state is closely related to adipose tissue hypoxia (3). A pivotal step to becoming obese is the rapid expansion of adipose tissue (AT) (4). The progression is accompanied by the shortage of oxygen due to the inability of the vascular system to keep up with the massive expansion. Hypoxia is therefore emerging as a causative factor for adipose dysfunction in obesity (4). The hypoxia state in AT induces the accumulation of hypoxia-inducible factor 1α (HIF-1α), which regulates many cellular anti-hypoxic responses (5, 6). Evidence from many laboratories suggests that the oxygen shortage of AT leads to several pathological processes, such as fibrosis, adipocytokine dysregulation, and inflammation, which are closely associated with adipose dysfunction and metabolic disorders (3, 6, 7).

HIF1α is considered as a significant initiating factor in adipocytes for fibrotic and inflammatory response, directly linked to metabolic dysfunction in AT under hypoxic conditions (3, 8). Unlike its action in the tumor, HIF1α cannot promote an adaptive proangiogenic reaction in AT (8). Instead, HIF1α induces an alternative transcriptional program, mainly entailing enhanced synthesis of extracellular matrix (ECM) components (9). The excessive collagen deposition in AT impairs ECM flexibility and tissue plasticity, thereby limiting AT’s overexpansion, which triggers adipocyte necrosis (4, 8). The proinflammatory M1 macrophages are recruited to remove dead adipocytes, which eventually results in inflammation (10). HIF1α may also directly upregulate proinflammatory factors, such as IL-6 and MIF. These proinflammatory factors increase the infiltration of M1 macrophages, which in turn causes adipose fibrosis. These events raise the possibility that suppression of HIF-1α can prevent fibrotic and inflammatory changes induced by hypoxia or obesity (11, 12).

Nicotinamide mononucleotide (NMN), a member of the vitamin B_3_ family, is a defined biosynthetic precursor of nicotinamide adenine dinucleotide (NAD^+^). The effect of NMN on restoring NAD^+^ homeostasis and activating sirtuins has been extensively investigated in the past decades (13, 14). Through restoring NAD^+^ homeostasis, NMN can regulate mitochondrial metabolism and oxidative signaling pathway, which are closely related to adipose inflammation and fibrosis. NMN can also activate sirtuins, known to be the key regulators of aging and longevity, which have been linked to the regulating of adipose tissue function, including adipogenesis, WAT inflammation, and adipokine secretion (15–17). For example, SIRT1, the most well-known and prevalent sirtuin, has been reported to inhibit adipose inflammation. Genetic Sirt1 deficiency in rodents leads to elevated inflammation characterized by increased macrophage infiltration and mRNA expression of inflammatory cytokines in WAT under the High Fat Diet (HFD) condition (17–19). Moreover, SIRT1 has been involved in cardiac and renal fibrosis by regulating the TGF-β/Smads signaling pathway (20, 21). However, few studies have investigated whether SIRT1 inhibits hypoxia-induced inflammation and fibrosis by regulating HIF1α signaling pathway, especially in WAT. On the other hand, NMN has entered the stage of preclinical research due to its fewer unfavorable side effects and higher orally bioavailable (22). It has been well-documented that NMN delayed aging and ameliorated diseases caused by NAD^+^ depletion, such as Alzheimer’s disease, heart failure, diabetes, and complications associated with obesity (23–25). A recent study has shown that one adaptive metabolic pathway mediated by nicotinamide phosphor-ribosyl-transferase (NAMPT, the rate-limiting enzyme in NAD^+^ biosynthesis) and SIRT1 is severely compromised in white adipose tissue (WAT) by HFD, thus leading to diabetes, whereas NMN ameliorates these changes (26). Although several preliminary studies have reported the effect of NMN on diet-and age-induced metabolic dysfunction, little is known about its role in hypoxia induced adipose fibrosis and inflammation.

In the present study, we aimed to find out: (a) whether NAMPT/NAD^+^/SIRT1 axis is compromised by hypoxia, thus leading to adipose fibrosis and inflammation; (b) whether NMN inhibits hypoxia-related adipose fibrosis and inflammation with an emphasis on regulating HIF1α by restoring the compromised NAD^+^/SIRT1 axis.

## Methods

### Chemical and reagents

Nicotinamide mononucleotide (B7878) was purchased from APExBIO Technology (Houston, United States) (98% purity, as detected by high-performance liquid chromatography analysis). Sirtinol was obtained from MedchemExpress (Sollentuna, Sweden). CoCl_2_ was obtained from Sigma-Aldrich Co (St. Louis, MO, United States). The reverse-transcription assay kit was obtained from Thermo Fisher Scientific (USA). Antibodies against the following proteins were obtained from Abcam: TGF-β1 (ab92486), SIRT1 (ab110304), adiponectin (APN, ab5694), and NAMPT(ab236874). Antibodies against HIF1α (NB100-105) were purchased from Novus Biologicals. Antibodies against acetyl-lysine (9441S) were purchased from Cell Signaling Technology. Antibodies against MMP9(27306-1-AP), TIMP1(16644-1-AP), IL-6 (66146-1-lg), GAPDH, and β-actin were obtained from the Proteintech Group.

### Animal experiments

Male C57/B6 mice (8-10 weeks) weighing 20.2 ± 2g were purchased from the Institute of Laboratory Animal Science, Hunan SJA Laboratory Animal Co., Ltd (Changsha, China). All animal experiments were under the approval of the Animal Care and Use Committee of Second Xiangya Hospital of Central South University and were performed in strict accordance with the recommendations in the Guide for the Care and Use of Laboratory Animals of the National Institutes of Health. Mice were housed in a specific-pathogen-free (SPF) environment with a regular 12/12h day/night cycle, a humidity of 70%, and a temperature of 22°C for seven days before experiments. Mice were randomly divided into three groups (n = 8), namely, Control, hypoxia, and hypoxia + NMN. Hypoxia group mice were placed in a hypoxic chamber (54.02 kPa, 10.8% O_2_) for four weeks. The chamber was opened daily for 30 min to clean and replenish food and water. NMN (500mg/kg, i.p, every three days) was delivered to mice 5-days before hypoxic treatment and one week after hypoxic treatment finished (25). The mice in the hypoxia and control groups were delivered equal volumes of saline. After anesthetic induction, the mice are sacrificed by cervical dislocation. Lay the mouse in a supine position. Secure the upper and lower limbs to the dissection pan. Remove the skin, locate the testes and use forceps to lift up the epididymal white adipose tissue (eWAT). Use iris scissors to carefully excise the WAT from the epididymis. Fix one part of eWAT in 10% neutral buffered formalin for 24 h prior to histological processing and store the rest in a -80°C refrigerator.

### Histological study

After overnight fixation in 4% paraformaldehyde, the obtained eWAT was embedded in paraffin and sliced into 5-μm-thick sections. The change in eWAT structure and adipocyte size was examined by hematoxylin and eosin (H&E)-stained sections. The extent of interstitial fibrosis in WAT was evaluated from Masson-stained sections. It was calculated as the mean ratio of the blue-stained fibrotic area to the total tissue area. For each section, five optical fields were analyzed using digital analysis software (Image J) in a blinded manner.

We performed immunohistochemical staining to assess the level of SIRT1 expression and macrophage infiltration in the eWAT. After the sections were blocked using 8% goat serum in phosphate buffer saline (PBS), they were incubated with primary antibody against SIRT1 or the macrophage marker F4/80 at four °C overnight. Next, the sections were incubated with GT VisionTM+Detection System/Mo&Rb reagent for one hour at room temperature and developed using a peroxide-based substrate diaminobenzidine (DAB) kit (Gene Tech, Shanghai, China). Eventually, we dehydrated and cleared these sections in ethanol and xylene, respectively, and took the fields of view at the magnification of 200 ×. For each adipose depot, five optical fields per section were analyzed.

### Primary cell culture from the stromal vascular fraction of adipose tissue

Stromal vascular fraction (SVF) cells in epididymal fat depots from male C57BL/6 mice were isolated and cultured as described previously (27, 28). Briefly, fat tissues were minced into one mm^3^ piece, then digested by type I collagenase (1 mg/ml) under agitation for 30-40 min at a 37 °C water bath. SVF cells were separated from the top layer of mature adipocytes by centrifugation (700 g, 10 min), then suspended in DMEM/F12 (Gibco) with 10% FBS and 100 U/ml penicillin-streptomycin and filtered by the cell strainer. Next, we centrifuged and re-suspended the SVF pellets in fresh media. Two hours after culturing cells under normal conditions (at 37°C in 95% O_2_ and 5% CO_2_), we washed the cells with PBS twice to remove red blood cells, immune cells, and other contaminants, and fresh media were added. All cells between 3-5 passages were used in this experiment. After serum starvation for 24 h, cells were randomly placed in cell culture chamber with 1% O_2_ for 24h to conduct hypoxia. NMN was dissolved in sterile PBS and diluted to the desired final concentrations (200µM). NMN was added simultaneously with hypoxia treatment. Sirtinol (25µM) was used to block SIRT1 expression in SVFs. CoCl_2_ (100μM) was used to enhance HIF1α expression in SVFs. Sirtinol or CoCl_2_ was also added simultaneously with NMN.

### Western blot and real-time quantitative PCR

Immunoblotting analysis and qPCR were performed according to previous articles (29). In brief, Protein-extracts of snap-frozen eWAT and whole-cell lysates of SVF were prepared using standard procedures. Protein concentrations in the supernatants were measured using Bicinchoninic acid (BCA) assay (ASPEN, USA). Proteins were separated on SDS-polyacrylamide gels and transferred to PVDF membranes. After blocking with QuickBlock™ Western (P0252, Beyotime Biotechnology, China), the membranes were incubated with the primary antibodies overnight at 4°C, washed in PBST three times, and incubated with a secondary goat anti-rabbit polyclonal antibody (SA00001-2, Proteintech Group) at room temperature for 1h. Finally, the signals were tested by WesternBright™ Sirius ECL kit (K-12043-D20, Advansta, USA). Protein expression levels were normalized to β-actin.

Total mRNA was extracted from eWAT or SVF cells with GeneJET RNA Purification Kit (K0731, Thermo Fisher Scientific, USA). Reverse transcribed into cDNA using RevertAid First strand cDNA Synthesis kit (K1622, Thermo Fisher Scientific, USA). The StepOne Real-Time PCR (Life tech, Alameda, CA) was used for real-time qPCR analysis. The primers used are described in **Table S1**. β-actin was used as an internal control. The relative expression quantity 2^-ΔΔCt^ value was calculated to compare the differences among groups.

### Immunoprecipitation

For immunoprecipitation experiments, total homogenates from adipose tissue and cultured cells were treated with RIPA lysis buffer (P0013B, Beyotime Biotechnology, China), vortexed for the 30s, and centrifuged for 15 min at 12000 r/min. The tissue or cell extracts was subjected to immunoprecipitation with HIF1α primary antibody at 4°C overnight. The antibody-bound proteins were precipitated with 20 μL protein A/G PLUS-Agarose (Santa Cruz Biotechnology, sc-2003) and rotated for 1 h, then incubated overnight at 4°C. The beads were then gently centrifuged at 1000 r/min for 5 minutes at 4°C. After four RIPA buffer washes, the immunoprecipitates were diluted with 40 μL of 1 × SDS loading buffer (CW0027, Cowin Biotech, China) and boiled at 100°C for 2-3 min to separate complexes from the protein A/G PLUS-Agarose. The samples were then subjected to SDS-PAGE and transferred to polyvinylidene difluoride (PVDF) membranes (Bio-Rad, USA). After blocking with QuickBlock™ Western (P0252, Beyotime Biotechnology, China), the membranes were incubated with an anti-acetylated-lysine antibody (Cell Signaling Technology, #9441) overnight at 4°C, washed in PBST three times, and incubated with a secondary goat anti-rabbit polyclonal antibody (SA00001-2, Proteintech Group) at room temperature for 1h. Finally, the signals were tested by WesternBright™ Sirius ECL kit (K-12043-D20, Advansta, USA).

### NAD^+^ measurements

In order to measure the NAD^+^, EnzyChrom NAD^+^/NADH Assay Kit (ECND100, Bioassay Systems, Hayward, California) was used according to the manufacturer’s instructions. In brief, mice eWAT weighing 20 mg for each sample or cells pelleted about 10^5^ for each sample were taken and homogenized in 100 μL NAD^+^ or NADH extraction buffer, respectively. Extracts were heated for 5 min at 60°C, and 20 μL of assay buffer was added into extracts, followed by 100 μL of the opposite extraction buffer (to neutralize the extracts). Mixtures were vortexed and centrifuged at 12,000 g for 5 min. Supernatants (40 μL) were then mixed with a working reagent (80 μL) in each well. The optical density of supernatants at 565nm were measured at 0 and 15min intervals using a 96-well plate reader spectrophotometer. NAD^+^/NADH concentration and their ratio were calculated using the manufacturers’ equation.

### Statistical analysis

Statistical analyses were performed using GraphPad Prism version 7.0 software (San Diego, CA, USA). All values are expressed as mean ± standard deviation of the mean (SD). If the data fit normal distribution by taking Kolmogorov-Smirnov tests, the statistical comparisons between two groups were performed using Student’s t-test, and comparisons among multiple groups were performed using one-way ANOVA followed by Tukey’s *post hoc* test. *P <* 0.05 was considered statistically significant.

## Results

### NMN inhibits the aberrant deposition of ECM in the eWAT of hypoxia-induced mice

**Figure 1:**
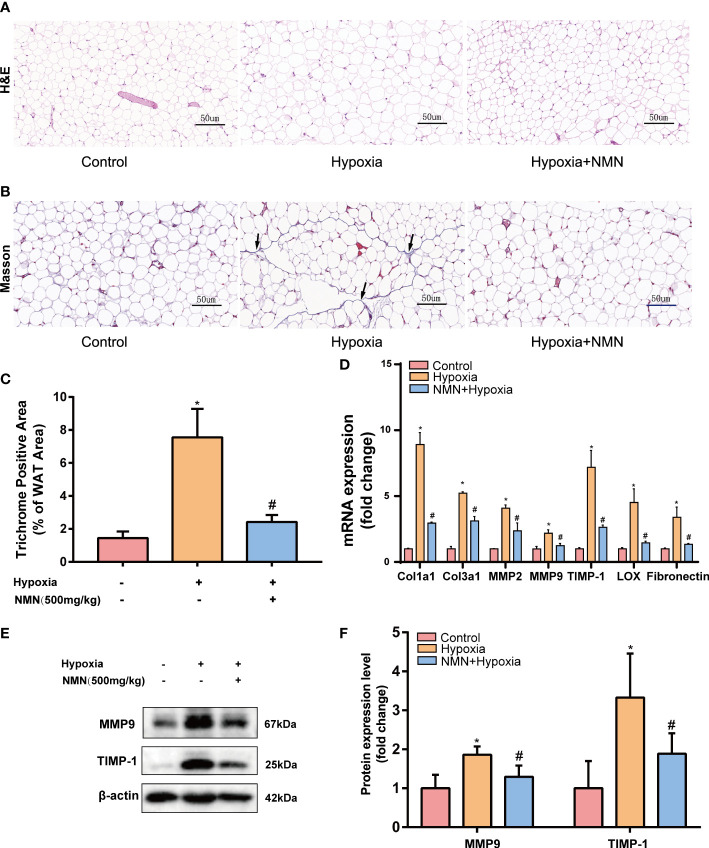
NMN alleviates the abnormal degradation and synthesis of ECM components in the eWAT of hypoxia-induced mice. **(A, B)** Representative images of eWAT adipocytes morphology and fibrosis as reflected by the H&E staining and Masson staining (400×magnification, scale bar=50 μm). **(C)** Statistical result for the interstitial fibrosis of eWAT (n = 6). **(D)** The relative mRNA levels of Col1a1, Col3a1, MMP-2, MMP-9, TIMP-1, LOX, and FN normalized to β-actin in eWAT (n = 3). **(E, F)** The relative protein levels of MMP9 and TIMP1 normalized to β-actin in eWAT (n = 5). *P < 0.05 versus control group, ^#^ *P* < 0.05 versus hypoxia group.

Adipose tissue structure remodeling is closely related to fibrosis. To learn whether NMN inhibits hypoxia induced adipose tissue structure change, we detected the adipocytes morphology and fibrotic area of the eWAT by HE Staining and Masson staining respectively (**Figures 1A, B**). Collagen fibers from the hypoxia-induced eWAT were increased and mainly distributed around adipocytes compared with the control group (7.556 ± 0.703 *vs.* 1.445 ± 0.1626, *P<0.05*). In contrast, the observed abnormal collagen deposition was reduced in NMN-treated eWAT (2.421 ± 0.1732 *vs.* 7.556 ± 0.703, *P<0.05*) (**Figures 1B, C**). Next, we measured the mRNA levels of collagen type I (Col1a1), type III (Col3a1), as well as matrix metalloproteinase 2 (MMP-2), MMP-9, tissue inhibitors of MMPs (TIMP-1), lysyl oxidase (LOX), and fibronectin (FN) by real-time quantitative PCR. As shown in **Figure 1D**, the above fibrotic genes were significantly upregulated in hypoxia-induced eWAT, whereas NMN reversed these changes. Moreover, the reduction of MMP9 and TIMP-1 protein expression levels due to NMN administration was further confirmed by Western blot (**Figures 1E, F**).

### Effect of NMN on dysregulated adipokines secretion and macrophage infiltration in the eWAT of hypoxia-induced mice

**Figure 2:**
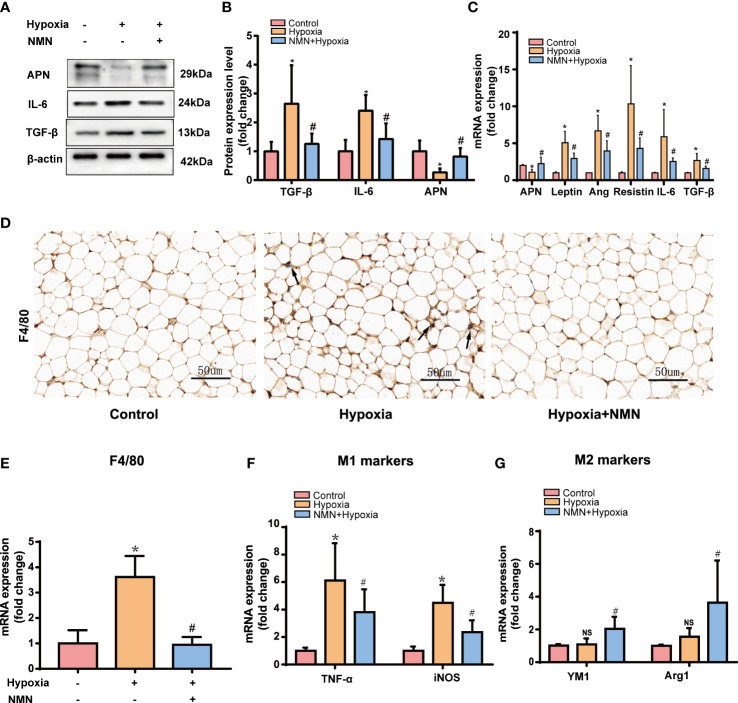
NMN alleviates dysregulated adipokines secretion and macrophage infiltration in the eWAT of hypoxia induced mice. **(A, B)** The relative protein levels of APN, TGFβ, and IL-6 normalized to β-actin in eWAT (n = 5). **(C)** The relative mRNA levels of APN, leptin, ang, resistin, IL-6, and TGF-β normalized to β-actin in eWAT (n = 6). **(D)** Representative images of macrophage marker F4/80 in eWAT as reflected by the IHC staining (400 × magnification, scale bar = 50μm). **(E)** The relative mRNA levels of F4/80 in eWAT (n = 6). **(F, G)** The relative mRNA levels of genes encoding TNFα, iNOS, Arg1 and Ym1 in eWAT (n = 6). *P < 0.05 versus control group, ^#^ *P* < 0.05 versus hypoxia group.

WAT is an active endocrine organ that produces many adipokines related to adipose dysfunction, especially the inflammatory and fibrotic factors. Therefore, we detected the protein level of APN, IL-6, and TGF-β in the eWAT of each group by Western blot. Hypoxia significantly increased pro-inflammatory and profibrotic factors levels, including IL-6 and TGF-βand decreased protective adipokine level, such as APN, whereas NMN attenuated these changes. (**Figures 2A, B**). The mRNA levels of the adipokines, including Leptin, Ang, Resistin, IL-6, and TGF-β, were significantly higher in the hypoxia group while APN expression was decreased (**Figure 2C**); NMN reversed these changes of the above adipokines.

We next examined the expression of macrophage marker F4/80 by immunohistochemistry (IHC) and qPCR. NMN significantly reduced the amounts of macrophages in the eWAT (**Figures 2D, E**). To further assess the effect of NMN on macrophage polarization in the eWAT, we evaluated the mRNA levels of TNFα and iNOS (M1 phenotype markers) and two other proteins, Arg1 and Ym1 (M2 phenotype markers). Compared with the hypoxia group, mice treated with NMN had lower levels of TNFα and iNOS but higher Arg1 and Ym1 levels (**Figures 2F, G**).

### NMN restores NAMPT/NAD+/SIRT1 axis and inhibits HIF-1α in white adipose tissue of hypoxia-induced mice

**Figure 3:**
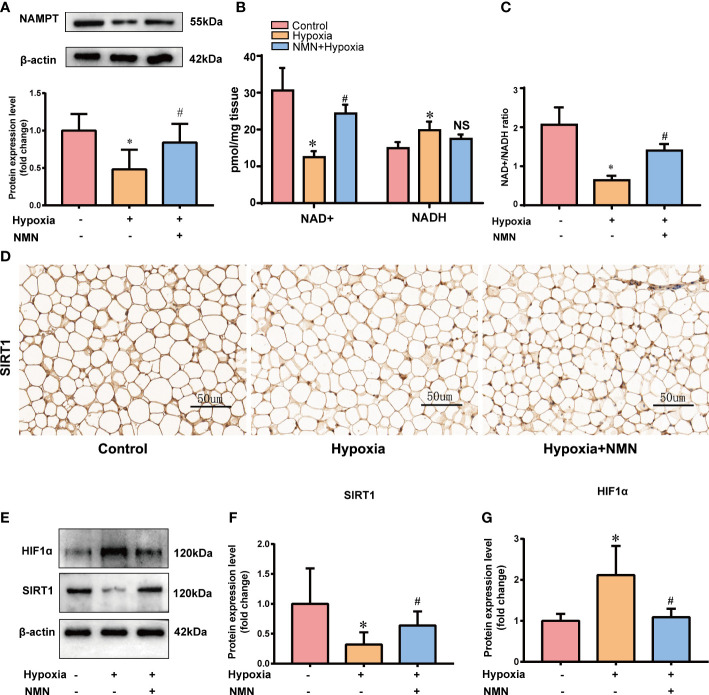
NMN restores NAMPT/NAD+/SIRT1 axis and inhibited HIF-1α acetylation in eWAT of hypoxia induced mice. **(A)** The relative protein levels of NAMPT normalized to β-actin in eWAT (n = 5). **(B, C)** Statistical results for NAD^+^/NADH contents and NAD^+^/NADH ratio in eWAT (n = 6). **(D)** Representative images of immunohistochemical staining for the SIRT1 protein in eWAT sections (400× magnification, scale bar=50μm). **(E–G)** The relative protein levels of SIRT1 and HIF1α in eWAT (n = 5). *P < 0.05 versus control group, ^#^ *P* < 0.05 versus hypoxia group. NS indicates no significant difference compared with the matched group.

As a biosynthetic precursor of NAD^+^, NMN was reported to boost the NAD^+^ pool *in vivo* and regulate its related pathway. Thus, we evaluated NAMPT/NAD+/SIRT1 axis in the eWAT of three groups. The protein levels of NAMPT were significantly decreased in the eWAT of hypoxia-induced mice, and the impairment was restored by NMN(**Figure 3A**). NAD^+^ levels and the NAD^+^/NADH ratio in eWAT were reduced by hypoxia treatment but were replenished by NMN (**Figures 3B, C**). We next examined the protein expression of SIRT1 in the hypoxia group by IHC and Western blot (**Figures 3D–F**). It was significantly decreased in hypoxia-induced eWAT and was notably increased by NMN. HIF-1α is a key regulator in hypoxia-induced eWAT fibrosis. Thus, we examined the effects of NMN on HIF-1α in the eWAT of three groups (**Figures 3D, G**) and found it can diminish HIF-1α expression in hypoxia.

### NMN suppresses HIF-1α signaling-associated upregulation of fibrogenic and inflammatory genes in a SIRT1-dependent manner *in vitro*.

**Figure 4:**
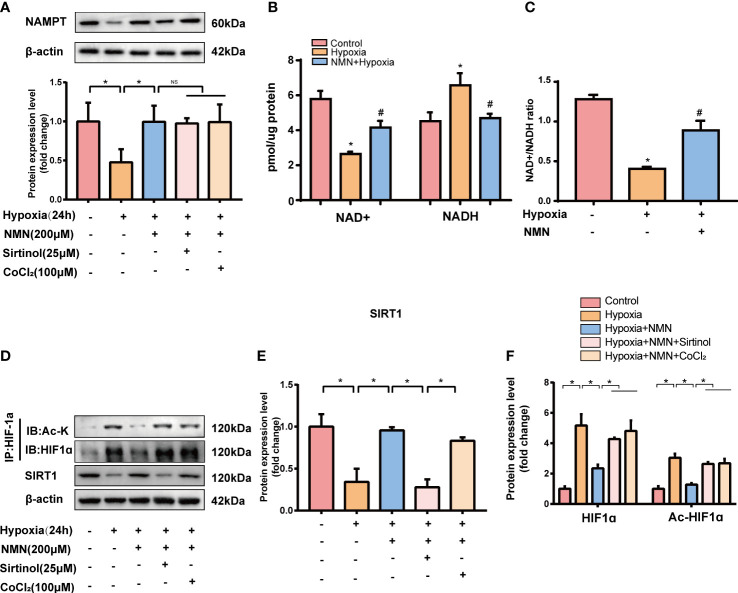
NMN restores NAMPT/NAD+/SIRT1 axis and inhibited HIF-1α acetylation in SVF cells. **(A)** The relative protein levels of NAMPT normalized to β-actin in SVF cells (n = 3). **(B, C)** Statistical results for NAD^+^/NADH contents and NAD^+^/NADH ratio in SVF cells (n = 3). **(D–F)** The relative protein levels of SIRT1 and HIF1α in SVF cells (n = 3). *P < 0.05 versus control group, ^#^ *P* < 0.05 versus hypoxia group. NS indicates no significant difference compared with the matched group.

To further assess whether the inhibitory effect of NMN on eWAT structure remodeling is dependent on SIRT1 and its possible downstream target-HIF1α, SVF cells isolated from eWATof lean mice were exposed to hypoxia with or without NMN (200µM), Sirtinol (a SIRT1 inhibitor, 25µM) and CoCl_2_ (a HIF1α enhancer, 100μM). The protein levels of NAMPT were significantly decreased in hypoxia-induced SVF cells, and NMN restored the impairment independently of Sirtinol or CoCl_2_ treatment (**Figure 4A**). NAD^+^ levels and the NAD^+^/NADH ratio in SVF cells were reduced by hypoxia treatment but were replenished by NMN (**Figures 4B, C**). SVF cells showed a significantly hypoxia-induced downregulation of SIRT1 and upregulation of HIF1α and ac-HIF1α (**Figures 4D–F**). NMN treatment reversed the above changes. Interestingly, sirtinol partially blocked the activation of SIRT1 by NMN, accompanied by increased HIF1α and ac-HIF1α. CoCl_2_ also augmented the level of HIF1α and ac-HIF1α after NMN treatment (**Figures 4D, F**).

**Figure 5:**
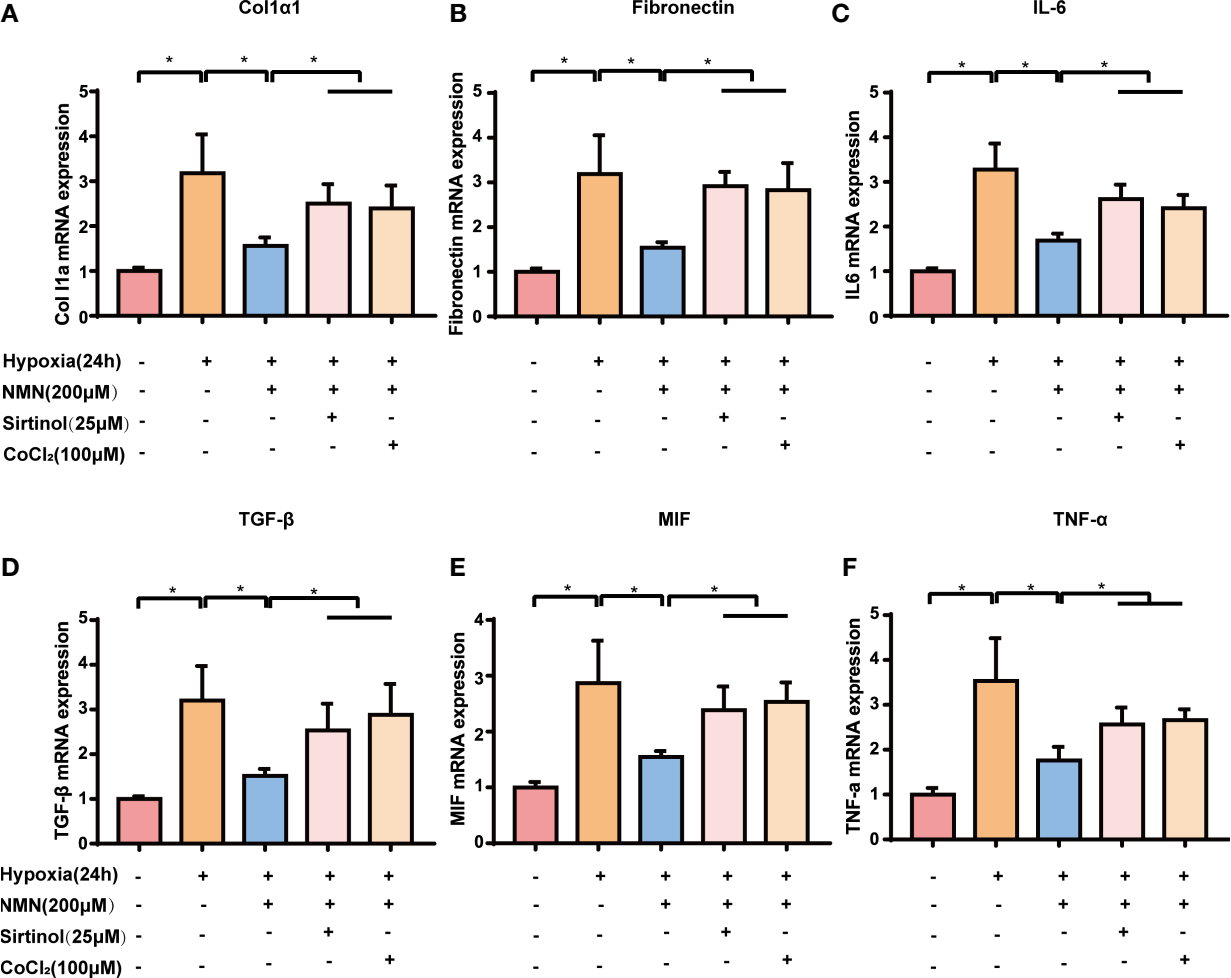
NMN suppresses HIF-1α signalling-associated upregulation of fibrogenic and inflammatory gene in a SIRT1-dependent manner. **(A–F)** The relative mRNA levels of Col1α, Fibronectin, IL-6, TGF-β, MIF and TNF-α normalized to β-actin in SVF cells (n = 3). *P < 0.05 versus control group, ^#^ *P* < 0.05 versus hypoxia group.

We next examined the expression of fibrogenic and inflammatory genes associated with HIF1α signaling in SVF cells, including Col1a1, FN, TGF-β, IL-6, MIF, and TNF-α. The expression of the above genes was significantly increased in hypoxia-induced SVF cells, and this effect was reversed by NMN (**Figures 5A–F**). However, the suppressive effects of NMN on these genes were partly abolished by sirtinol (a SIRT1 inhibitor) or CoCl2 (a HIF1α enhancer).

## Discussion

Adipose tissue hypoxia and the activation of HIF-1α in obesity contribute to insulin resistance and type 2 diabetes. The mice raised in the hypoxia chamber presented similar pathophysiological processes, such as adipose fibrosis and inflammation, compared with HFD-fed mice (3, 12, 30). Recently, NAD^+^ depletion is emerging as a major contributor to the pathogenesis of metabolic diseases, with the rise of studies on NAD^+^ repletion strategies as countermeasure (14). In obesity, one of the main factors causing NAD^+^ depletion in AT is that energy or fat excess, such as HFD-feeding, inhibits NAMPT expression (26, 31, 32). However, whether hypoxia is involved in the compromised NAMPT-mediated NAD^+^ biosynthesis, thus leading to adipose fibrosis and inflammation is unknown. We previously demonstrated that NMN attenuates the development of cardiac fibrosis by inhibiting oxidative stress and the TGF-β/Smad signaling pathway, but whether NMN plays a role in adipose fibrosis and its mechanism is dependent on HIF1α are unclear. To fill this knowledge gap, we explored the effect of NMN on hypoxia-induced NAD^+^ metabolism, fibrosis, and inflammation in the eWAT of mice. In the present study, hypoxia- induced NAMPT down-regulation, NAD^+^ depletion, fibrosis, and inflammation in eWAT, whereas the NAD^+^ precursor NMN attenuated the changes, indicating the involvement of NAD^+^ depletion in hypoxic insult. NMN attenuated HIF1α expression, suggesting that NAD^+^ depletion may contribute to the HIF-1α accumulation in response to hypoxia. NAD^+^ depletion and HIF-1α accumulation are the stress response to AT hypoxic state in the long term. In this context, the inhibitory role of NMN in NAD^+^ depletion and hypoxia in AT was an attempt to restore cellular homeostasis.

The induction of HIF-1α in a hypoxia state fails to activate the angiogenic program in AT and turns to initiate a profibrotic response instead, causing fibrosis and inflammation in adipose tissue (4, 8). Inhibition of HIF -1α is the key point to attenuating adipose fibrosis and inflammation. In the present study, we found that NMN can inhibit HIF-1α activation and, as a result, effectively inhibit these profibrotic gene expressions. Consistent with the above results, the reduced Masson’s staining further confirmed the anti-fibrotic effects of NMN. In addition, excessive collagen accumulation in AT is closely related to inflammation characterized by inflammatory cytokines dysregulation and macrophage infiltration (7), and HIF-1α can induce NF-κB-dependent inflammation (7, 33). In the AT of hypoxia-induced mice, NMN decreased the pro-inflammatory cytokines (TNF-α, TGF-β, and IL-6) and deleterious adipokines secretion (Leptin and Resistin) but increased the expression of adiponectin (APN). APN is a vital adipokine with insulin-sensitizing and anti-inflammation activities (34–36), but hypoxia could potentially inhibit APN and subsequently lead to inflammation and M1 macrophage infiltration in AT (37, 38). The anti-inflammatory activity of NMN may also be partly attributed to the regulation of APN. In the adipose tissue of hypoxia mice, NMN not only reduced the F4/80 (an indicator for the amounts of macrophage) but also inhibited M1 macrophage polarization, indicating the effect of NMN on blocking the inflammatory interplay between macrophages and adipocytes. The above evidence showed the protective role of NMN aganist adipose fibrosis and inflammation may be associated with the inhibition of HIF -1α.

NMN has been reported to be a multifunctional compound. In addition to replenishing NAD^+^ and inhibiting oxidative stress, it can activate SIRT1, contributing to its effects on fibrosis and inflammation (21, 26, 39). In our *in vitro* study, the anti-fibrotic and anti-inflammatory effects of NMN on hypoxia-induced SVF were blocked by Sirtinol (a SIRT1 inhibitor), indicating the involvement of SIRT1 in NMN treatment. As an energy sensor, SIRT1 regulates cellular homeostasis, and therefore, we wanted to know whether NMN inhibited HIF-1α accumulation by activating SIRT1. Gomes et al. have found that SIRT1 is constantly required to ensure the efficient degradation of HIF1α under normal oxygen condition (40). During aging or hypoxia, NAD^+^ depletion reduces the activity of SIRT1, leading to the activation of HIF1α (40, 41). Consistently, whether *in vivo* or *in vitro*, our results showed that the hypoxia-induced HIF1α upregulation was accompanied by reduced NAD^+^ levels and SIRT1 activity. NMN treatment failed to reverse the above process with the disturbance of Sirtinol, indicating that NMN reduced HIF-1α accumulation by promoting proteasomal degradation in a SIRT1-dependent manner. Although the HIF-1α stabilizer CoCl_2_ treatment can enhance the HIF1α expression with the existence of NMN, it failed to block the activation of SIRT1, further demonstrating that HIF-1α is the downstream target of SIRT1. In addition, several studies have shown that SIRT1 inactivated HIF1α by deacetylating it and consequently inhibited HIF-1 target genes (41, 42). In the present study, Sirtinol attenuated the enhanced effect of NMN on HIF1α deacetylation, indicating a possible role of SIRT1 in HIF1α activation. The above evidence is probably why SIRT1 has a role in the effect of NMN on HIF1α.

**Figure 6:**
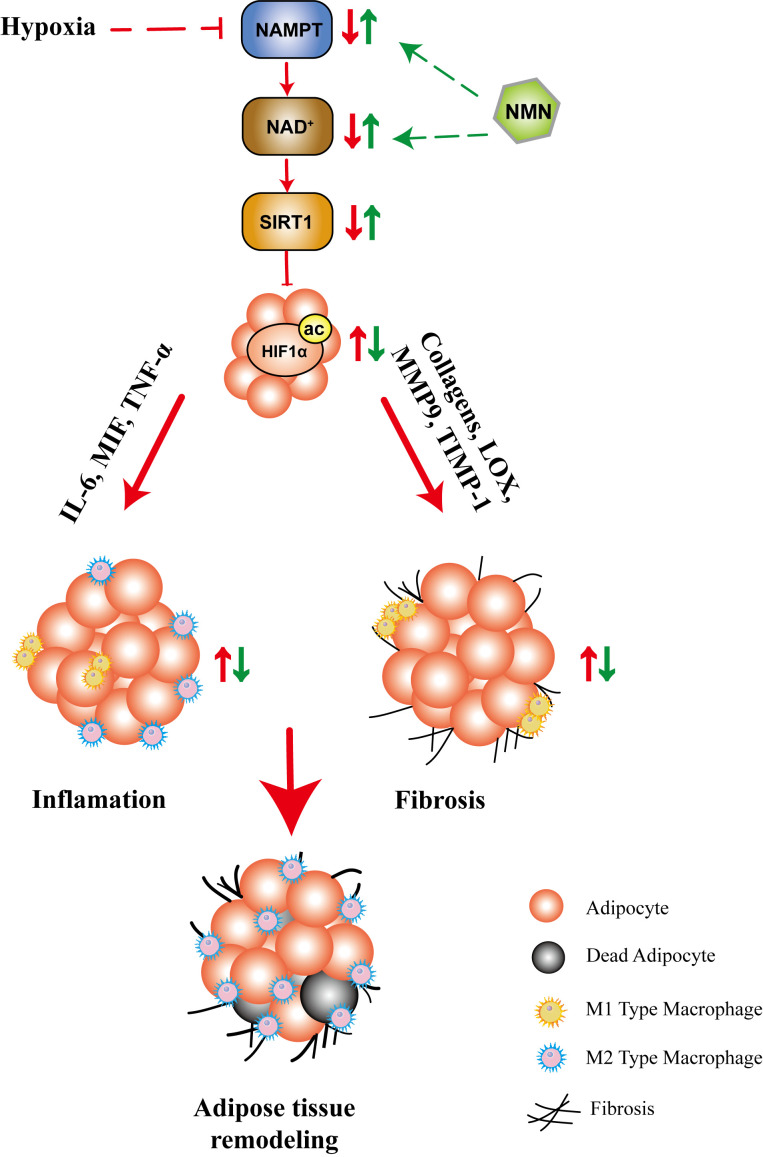
The proposed mechanisms for the protective role of NMN in hypoxia-induced adipose tissue remodeling. Hypoxia led to compromised NAMPT/NAD^+^/SIRT1 axis, which further promoted HIF1α activation. As a result, the fibrotic and inflammatory response are significantly increased, accompanied by adipocytokine dysregulation, which ultimately lead to adipose tissue remodeling. NMN restored the NAMPT/NAD^+^/SIRT1 axis and inhibited HIF1α activation, which further attenuated adipose tissue remodeling.

In summary, our study showed that NMN inhibited HIF-1α activation-induced adipose tissue fibrosis and inflammation, while the underlying mechanism may be associated with the NAD^+^ repletion and the regulation of SIRT1 (**Figure 6**). These results provided further evidence for the beneficial effects of NMN on regulating adipose function in hypoxia. The benefit of NAD precursors in mouse model of metabolic diseases, in particularly NMN and NR, has been extensively investigated for a long time (23, 26, 39, 43). Currently, there are several ongoing human clinical trials or recently reported trials (NCT02191462, NCT02689882, NCT02921659, NCT02303483, NCT02678611, NCT03151239, UMIN000021309, UMIN000025739, UMIN000030609). All reported clinical trials of nicotinamide riboside (NR) or NMN demonstrated that it is safe, well tolerated, and can significantly increase plasma NAD^+^ levels in healthy or obese volunteers (44–48). Besides, a recent study showed that 10 weeks of NMN administration in doses of 250mg/d improved skeletal muscle insulin sensitivity and insulin signaling in women with prediabetes (48). As NAD^+^ metabolism can be a potential target for pathophysiological processes, including mitochondrial metabolism, oxidative stress, inflammation, and fibrosis, NMN or NR may become a therapeutic option. The above evidence increases the possibility of NAD^+^ precursors’ clinical application, but its efficacy in patients with metabolic disorders remains unclear, and more studies are needed.

## Data availability statement

The original contributions presented in the study are included in the article/**Supplementary Material**. Further inquiries can be directed to the corresponding authors.

## Ethics statement

The animal study was reviewed and approved by the Animal Ethical and Welfare Committee of Second Xiangya Hospital of Central South University.

## Author contributions

KW, BL, NL, and QML designed the research. KW and BL performed the experiments. KW was a major contributor in writing the manuscript. YM and BL revised the manuscript. QML and NL supplied financial support and revised the manuscript. All authors read and approved the final manuscript.
