# Supplementary material for: Nicotinamide mononucleotide attenuates HIF-1α activation and fibrosis in hypoxic adipose tissue via NAD+/SIRT1 axis
Source: Front Endocrinol (Lausanne). 2023 Jan 26;14:1099134. doi: 10.3389/fendo.2023.1099134 (PMC9909340; doi:10.3389/fendo.2023.1099134)
Supplement: Supplementary file 1 [file Table_1.docx]

Supplementary Material

# Methods

## Supplementary Table 1. Sequences of primers used for real-time PCR

| Species | Genes | Sequences |
| --- | --- | --- |
| Mouse | β-actin | Forward: 5’-GACGTGGAACTGGCAGAAGAG-3’  Reverse: 5’-TTGGTGGTTTGTGAGTGTGAG-3’ |
| Mouse | TNF-α | Forward: 5’-TTCCTGCTGTTTCTCTTACACCT-3’  Reverse: 5’-CTGTCTGCCTCTTTTGGTCAG-3’ |
| Mouse | IL-6 | Forward: 5’-TAGTCCTTCCTACCCCAATTTCC-3’  Reverse: 5’-TTGGTCCTTAGCCACTCCTTC-3’ |
| Mouse | TGF-β1 | Forward:5’-CAGGAATGCACCAAGTACAAAGT-3’  Reverse:5’-CCTGGTCCAGTGAAGTTCAGC-3’ |
| Mouse | Col I α1 | Forward: 5’-CCCAACCCAGAGATCCCATT-3’  Reverse: 5’-GAAGCACAGGAGCAGGTGTAGA-3’ |
| Mouse | Col III α1 | Forward: 5’ -GATCAGGCCAGTGGAAATGT-3’  Reverse: 5’ -GTGTGTTTCGTGCAACCATC-3’ |
| Mouse | Fibronectin | Forward: 5’ -ATGTGGACCCCTCCTGATAGT-3’  Reverse: 5’ -GCCCAGTGATTTCAGCAAAGG-3’ |
| Mouse | MMP-2 | Forward: 5’ -CAAGTTCCCCGGCGATGTC-3’  Reverse: 5’ -TTCTGGTCAAGGTCACCTGTC-3’ |
| Mouse | MMP-9 | Forward: 5’ -CTGGACAGCCAGACACTAAAG-3’  Reverse: 5’ -CTCGCGGCAAGTCTTCAGAG-3’ |
| Mouse | LOX | Forward: 5’ -TCTTCTGCTGCGTGACAACC-3’  Reverse: 5’ -GAGAAACCAGCTTGGAACCAG-3’ |
| Mouse | TIMP-1 | Forward: 5’ -GCAACTCGGACCTGGTCATAA-3’  Reverse: 5’ -CGGCCCGTGATGAGAAACT-3’ |
| Mouse | Resistin | Forward: 5’ -AAGAACCTTTCATTTCCCCTCCT-3’  Reverse: 5’-GTCCAGCAATTTAAGCCAATGTT-3’ |
| Mouse | Angiotensin | Forward: 5’ -TCTCCTTTACCACAACAAGAGCA-3’  Reverse: 5’ -CTTCTCATTCACAGGGGAGGT-3’ |
| Mouse | APN | Forward: 5’ -TGTTCCTCTTAATCCTGCCCA-3’  Reverse: 5’ -CCAACCTGCACAAGTTCCCTT-3’ |
| Mouse | Leptin | Forward: 5’ -GAGACCCCTGTGTCGGTTC-3’  Reverse: 5’ -CTGCGTGTGTGAAATGTCATTG-3’ |
| Mouse | iNOS | Forward: 5’-GTTCTCAGCCCAACAATACAAGA-3’  Reverse: 5’ -GTGGACGGGTCGATGTCAC-3’ |
| Mouse | Ym1 | Forward: 5’ -CAGGTCTGGCAATTCTTCTGAA-3’  Reverse: 5’-GTCTTGCTCATGTGTGTAAGTGA-3’ |
| Mouse | Arg1 | Forward: 5’ -CTCCAAGCCAAAGTCCTTAGAG -3’  Reverse: 5’ -AGGAGCTGTCATTAGGGACATC-3’ |
| Mouse | F4/80 | Forward: 5’ -TGACTCACCTTGTGGTCCTAA-3’  Reverse: 5’ -CTTCCCAGAATCCAGTCTTTCC-3’ |

## Immunoprecipitation

For immunoprecipitation experiments, total homogenates from adipose tissue and cultured cells were treated with RIPA lysis buffer (P0013B, Beyotime Biotechnology, China), vortexed for 30s, and centrifuged for 15 min at 12000 r/min. The tissue or cell extracts was subjected to immunoprecipitation with HIF1α primary antibody at 4°C overnight. The antibody-bound proteins were precipitated with 20 μL protein A/G PLUS-Agarose (Santa Cruz Biotechnology, sc-2003) and rotated for 1 h to overnight at 4°C. The beads were then gently centrifuged at 1000 r/min for 5 minutes at 4 °C. After four RIPA buffer washes, the immunoprecipitates were diluted with 40 μL of 1 × SDS loading buffer (CW0027, Cowin Biotech, China) and boiled at 100°C for 2-3 min to separate complexes from the protein A/G PLUS-Agarose. The samples were then subjected to SDS-PAGE and transferred to polyvinylidene diﬂuoride (PVDF) membranes (Bio-Rad, USA). After blocking with QuickBlock™ Western (P0252, Beyotime Biotechnology, China), the membranes were incubated with an anti-acetylated-lysine antibody (Cell Signaling Technology, #9441) overnight at 4°C, washed in PBST three times, and incubated with a secondary goat anti-rabbit polyclonal antibody (SA00001-2, Proteintech Group) at room temperature for 1h. Finally, the signals were tested by WesternBright^TM^ Sirius ECL kit (K-12043-D20, Advansta, USA).

## NAD^+^ measurements

Mice fat tissue weighing 20 mg for each sample or cells pelleted about 10^5^ for each sample, were taken and homogenized in 100 μL NAD^+^ or NADH extraction buffer, respectively. Extracts were heated for 5 min at 60°C and 20 μL of assay buffer was added into extracts, followed by 100 μL of the opposite extraction buffer (to neutralize the extracts). Mixtures were vortexed and centrifuged at 12,000 g for 5 min. Supernatants (40 μL) were then mixed with a working reagent (80 μL) each well. Optical density of supernatants at 565nm were measured at 0 and 15min interval using a 96-well plate reader spectrophotometer. NAD^+^/NADH concentration and their ratio were calculated using the manufacturers’ equation.
